# Quantitative Proteomics Reveals That a Prognostic Signature of the Endometrium of the Polycystic Ovary Syndrome Women Based on Ferroptosis Proteins

**DOI:** 10.3389/fendo.2022.871945

**Published:** 2022-07-14

**Authors:** Jian Zhang, Nan Ding, Wenhu Xin, Xin Yang, Fang Wang

**Affiliations:** Department of Reproductive Medicine, Lanzhou University Second Hospital, Lanzhou, China

**Keywords:** ferroptosis, endometrial proteins, quantitative proteomics, prognostic model, PCOS

## Abstract

**Objective:**

We aimed to study the relationship between ferroptosis proteins and reproductive outcomes of infertile patients with PCOS and construct the related prognostic model.

**Methods:**

These endometrium samples of the study were collected from 33 women with PCOS and 7 control women with successful pregnancies at the Reproductive Center of Lanzhou University Second Hospital, September 2019 to September 2020. The 40 patients’ endometrium was identified the differentially expressed proteins (DEPs) using liquid chromatography tandem mass spectrometry. The Kyoto Encyclopedia of Genes and Genomes (KEGG) analysis and Gene Ontology (GO) showed that the DEPs related pathways and functions between PCOS and controls. Subsequently, univariate Cox regression analysis and Lasso regression were used to identifying independent prognostic ferroptosis proteins, which were utilized to establish a prognostic model. Then the performance of the prognostic model was evaluated by receiver operating characteristic curve (ROC) and decision curve analysis (DCA). Then clinical data and prognostic model were used to predict the reproductive outcomes of PCOS patients by constructing the nomograms. Finally, we performed the single sample gene set enrichment analysis (ssGSEA) to explore the correlation between risk scores and immune status.

**Results:**

A total of 5331 proteins were identified, 391 proteins were differentially expressed in the PCOS and controls. The KEGG analysis revealed that the ferroptosis pathway was significantly different between PCOS and controls. 5 ferroptosis proteins (GPX4, DPP4, G6PD, PCBP1, and PCBP2) prognostic model (FerSig) was constructed *via* Cox regression and Lasso regression. Patients were separated into high and low-risk groups according to the FerSig. Kaplan-Meier curve showed that patients in the low-risk group had much better reproductive outcomes than those in the high-risk group. The DCA showed that the risk score was an independent predictive factor for reproductive outcomes. Compared with clinical data, ROC curve analysis indicated the FerSig proteins as a potential diagnostic and prognostic factor in PCOS patients. Functional analysis revealed that the FerSig proteins and immune microenvironment were correlated to the prognosis of PCOS.

**Conclusion:**

The prognostic model focused on the FerSig proteins could predict the reproductive outcomes of PCOS patients with decreased endometrial receptivity, and provided theoretical basis for individualized treatment.

## Introduction

Polycystic ovary syndrome (PCOS) is the most frequent and complicated reproductive endocrine disease caused by endocrine and metabolism in women of reproductive age. It is primarily featured by hirsutism, acne, irregular menstruation, and infertility (1). In addition, although abnormal lipid metabolism (obesity, hyperlipidemia) and glucose metabolism (insulin resistance, hypertension) do not belong to the diagnostic criteria of PCOS, they are always associated with the occurrence and progression of PCOS (2–4). Globally, the prevalence of PCOS ranges between 6%-18%, and it is one of the main causes of infertility (5, 6).

PCOS diagnosis and treatment technology has advanced, but currently PCOS guidelines and consensus focus on treating and improving ovulation function rather than addressing issues of adverse fertility, such as implantation failure and abortion (7, 8). The mechanisms associated with implantation failure and abortion remain poorly uncertain (9–11). Adverse fertility has not been adequately addressed and resolved, and the prognostic risk of adverse fertility remains still high (12–14).

The main factor of adverse fertility is endometrial receptivity, which allows embryos to locate, adhere, and invade the uterine cavity (15). And reproductive outcomes were the best indicators of endometrial receptivity (16, 17). To help PCOS patients improve their endometrial receptivity, it is critical to investigate new factors in PCOS patients’ impaired endometrial receptivity. These factors will provide new concepts and strategies for the prevention and treatment of PCOS.

Studies conducted in the past few years have confirmed that mitochondrial dysfunction and increased oxidative stress are related to the progression and related complications in patients with PCOS (18–20). Oxidative stress based on increased ROS production can induce mitochondrial components impairment, such as mtDNA, proteins, and lipids (18, 21, 22). In addition, several antioxidant enzymes were reduced in PCOS clinical studies, such as superoxide dismutase (SOD), glutathione peroxidase (GPX), and catalase (CAT), and decreased the antioxidative capacity, suggesting that overexpression of ROS contributes to the progression of PCOS (23, 24). Oxidative stress induced ferroptosis is the basis of imbalanced redox homeostasis (25). Ferroptosis is a cell death characterized by increased iron content, lipid peroxidation, and plasma membrane damage (26, 27).

Little is known regarding the function of ferroptosis in PCOS infertility, as compared to apoptosis. Whether ferroptosis regulates endometrial receptivity in patients with PCOS is not yet established. This study aims to investigate the relationship between ferroptosis and endometrial receptivity and its effect on reproductive outcomes by using the PCOS patients’ endometrial proteomics and clinical prognostic data, providing a theoretical foundation for the treatment of improving PCOS endometrial receptivity.

## Materials and Methods

### Samples Collection

From September 2019 to September 2020, the endometrium samples were collected from 33 women with PCOS (aged 21–40 years old) and 7 control women with successful pregnancies at the Reproductive Center of Lanzhou University Second Hospital. PCOS patients who had undergone *in vitro* fertilization (IVF) were recruited as participants for our study. The patients who were recruited for the research had to satisfy all the following requirements simultaneously: (1) PCOS was diagnosed based on the Rotterdam criteria, which included two of three items: oligo- and/or anovulation, polycystic ovarian morphology on ultrasound, and hyperandrogenism. (2) the participants had no medication history during the previous 3 months before the initial diagnostic that confirmed PCOS; (3) the patients volunteered to participate in this clinical research and conformed well to this clinical study; (4) Married women with normal husband sperm. The exclusion criteria were the following: (1) Hormone drugs or drugs that affect insulin production were taken during the past 3 months before enrollment; (2) patients with cardiovascular disease, liver and kidney, hematopoietic system, and other diseases. The control group included non-PCOS women with successful pregnancies and live birth. The control patients had regular menstrual cycles and normal ovarian morphology *via* routine ultrasound scan. Informed permission was signed from all the participants before collecting samples. The study was authorized by the Ethics Committee of Lanzhou University Second Hospital (2017A-057).

**Figure 1:**
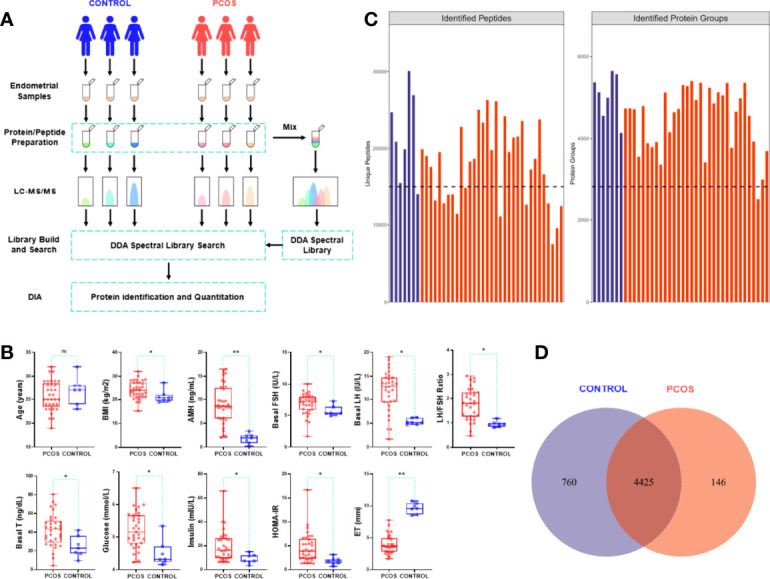
Protein identification analysis and clinical data evaluation.

The endometrial tissue specimens were taken from women with PCOS (n=33) and control (n=7). The endometrial samples were collected by the pipelle endometrial aspirator. The residual blood in the one part of endometrial tissue was cleaned with phosphate-buffered saline (PBS), and then put the cleaned sample into the cryopreservation tube and stored at -80°C for standby. The other part was examined by pathology. We then tracked the histological examination of endometrial specimens and selected that were taken during the proliferative stage. The samples collection and proteomic detection procession were shown in **Figure 1**.

### PCOS Clinical and Prognosis Data Collection

Age and body mass index (BMI) was recorded as the demographic characteristics. On the day of the endometrial biopsy, blood samples were examined for biochemical indicators, the anti-Müllerian hormone (AMH, reference range 1.22-11.70 ng/ml), and serum sex hormone concentrations. The biochemical indicators include serum lipids concentration, fasting plasma glucose, and insulin levels. Serum sex hormones include basal testosterone (T, reference range 8.40-48.10 ng/dl), basal luteinizing hormone (LH, reference range 2.40-12.60 mIU/ml), and basal follicle-stimulating hormone (FSH, 3.50-12.50 mIU/ml). The serum lipids examination includes cholesterol (TC, 2.30-5.80 mmol/L), triglycerides (TG, 0.45-1.80 mmol/L), high-density lipoprotein (HDL, 0.70-2.20 mmol/L), and low-density lipoprotein (LDL, 1.20-3.30mmol/L). The homeostasis model assessment insulin resistance (HOMA-IR) was calculated using the equation: fasting plasma glucose (reference range 3.60-6.10 mmol/L)×fasting insulin (reference range 3.00-25.00 mU/L)/22.5, and HOMA-IR ≥2.6 was considered to be IR (28). Endometrial thickness (ET) was examined by ultrasound scanning. All biochemical indexes and ultrasound scanning were tested in our hospital.

Reproductive outcomes and gestational duration were used as prognostic data for subsequent analysis. Reproductive outcomes were the best indicators of endometrial receptivity. Reproductive outcomes include live birth and adverse fertility (abortion and implantation). Gestational duration includes the gestational time of live birth and adverse gestational time weeks (including 4 weeks of no detected pregnancy and abortion) after IVF treatment. Gestational time was estimated in weeks.

### Protein Samples Preparation and Data-Dependent Acquisition Library Generation

The FastPrep-24 homogenizer (24×2ml tubes, mixing speed 6.0m/s, the 60s, twice) was used for protein extraction of the endometrial specimens. And then SDT buffer (4% SDS, 100 mM DTT, 150 mM Tris-HCl, pH=8.0) was added insolubilized in lysis. The lysates were further sonicated and boiled for 15 min. After centrifuging at 14000g for 40 min, the supernatant was estimated with the BCA Protein Assay Kit (Bio-Rad). Protein concentrations were estimated by the Bradford assay (Bio-Rad) and a mix of gamma globulin and human serum albumin was used as the standard. SDS-PAGE was performed by loading 200 μg protein from each sample in each well of 12% SDS polyacrylamide gels and then running the gels at 100 V for 2 h. The visualization of protein bands was accomplished *via* the application of colloidal Coomassie blue staining (**Supplementary Figure 1**). The sample was stored at -80°C. To generate a DDA library and ensure its quality, an equal aliquot of each sample was combined into one sample.

SDT buffer and an equal quantity of proteins for each sample were mixed well. The UA buffer (8M Urea, 150 mM Tris-HCl, pH=8.0) and ultrafiltration (Microcon units, 10kDa) were used to remove the detergent, dithiothreitol, and other low-molecular-weight components. The samples were then incubated in the dark for 30 minutes with 100 mL of iodoacetamide to prevent the reduction of cysteine residues. The filters were washed with UA buffer (100 μl, 3 times) and NH_4_HCO_3_ buffer (100 μl, 25mM, twice). 4 μg protease (Gibco) was used to digest the protein suspensions overnight at 37°C, and the produced peptides were collected as a filtrate. Each sample’s peptides were desalted on C18 Cartridges (3 ml volume, Sigma), concentrated in vacuum centrifugation, and reconstituted with formic acid (40 µl, 0.1%) to get peptides that could be used in further experiments. The peptide content was estimated by UV light spectral density at 280 nm. Digested pool peptides were then fractionated to 10 fractions using Thermo Scientific™ Pierce™ High pH Reversed-Phase Peptide Fractionation Kit. Each fraction was concentrated by vacuum centrifugation and reconstituted in formic acid (15 µl, 0.1%). Collected peptides were desalted on C18 Cartridges and reconstituted in formic acid (40 µl, 0.1%).

### Mass Spectrometry Assay for DDA and Data-Independent Acquisition

The DDA library creation required the injection of all fractions into the timsTOF mass spectrometer (Bruker) *via* an Evosep One system liquid chromatography. Protein samples were scanned with 8 cycles of parallel accumulation serial fragmentation (PASEF) MS/MS.

For DDA library data, the FASTA sequence database was searched with Spectronaut™ (Biognosys, version 14.4.200727.47784) software. The database was Uniprot_human database, iRT peptides sequence was added (Biognosys|iRTKit|). The parameters were set as follows: the enzyme was trypsin, max missed cleavages was 2, fixed modification is carbamidomethyl(C), dynamic modification is oxidation(M), and acetyl (Protein N-term). All reported data were based on 99% confidence for protein identification as determined by false discovery rate (FDR =N(decoy)*2/(N(decoy)+ N(target))) ≤ 1%. The spectral library was constructed by importing the original raw files and DDA searching results into Spectronaut Pulsar X™ (Biognosys, version 12.0.20491.4).

DIA data was analyzed with Spectronaut™ searching the above constructed spectral library. Main software parameters were set as follows: retention time prediction type is dynamic iRT, interference on MS2 level correction is enabled, and cross-run normalization is enabled. All results were filtered based on Q value cutoff 0.01 (equivalent to FDR<1%).

### Differential Protein Identification and Functional Enrichment Analysis

Briefly, the differential expression protein analysis was based on the R package (limma). Two criteria were used to identify Log fold change (LogFc) >0.585 and false discovery rate (FDR) adjusted *p*<0.05 were used as criteria to screen differentially expressed proteins.

The functional enrichment analysis was conducted using the R package (clusterProfiler). Subcellular localization (CELLO software version 2.5, http://cello.life.nctu.edu.tw/) of proteins was used to explore the functions of all differentially expressed proteins in cells. Gene Ontology (GO) enrichment analysis (including biological process, cellular component, and molecular function) and Kyoto Encyclopedia of Genes and Genomes (KEGG) pathway enrichment analysis were employed to further analyze the potential functions and molecular mechanism of all differentially expressed proteins based on the threshold with FDR<0.05 and *p*<0.05.

### Ferroptosis Relative Proteins Differential Analysis

Ferroptosis protein-coding genes sets were collected from FerrDb (http://www.zhounan.org/ferrdb/ ) and KEGG (https://www.kegg.jp/). 259 ferroptosis protein-coding genes were detailed in **Supplementary Table S1**. The ferroptosis differential proteins were screened *via* machine learning. The STRING database (version 11.5 https://cn.string-db.org/) was used to establish and visualize a protein-protein interaction (PPI) network of the ferroptosis differential proteins. The correlation analysis was used to visualize based on the expression of the ferroptosis differential expressed proteins (DEPs) R package (corrplot, reshape2, igraph).

### Establishment of the FerSig Risk Model

Based on the expression values of 10 above screened ferroptosis differential proteins, single factor Cox regression analysis was performed for PCOS samples, ferroptosis proteins significantly associated with prognosis data were screened with *p*<0.05. Then LASSO regression analysis with R package (glmnet) and seed 2000 was used to further identify ferroptosis proteins related to the prognosis of PCOS. The risk score outcome prediction was evaluated by using the ferroptosis signature (FerSig) formula as follows: $FerSig\;\left(PCOS\right)=\sum_{i=1}^{n}ceof\left(Ferpro_{i}\right)*expr\left(Ferpro_{i}\right)$ .*FerSig (PCOS)* represents a prognostic risk score, *ceof (Ferpro_i_)* represents the risk coefficient of ith prognostic ferroptosis protein. *expr (Ferpro_i_)* is the expression level of the ith prognostic ferroptosis protein for the patient. The PCOS samples were consequently separated by the risk score cutoff value (median risk score) of the low-risk or high-risk group by using the R package (survival, survminer). R package (survival and survminer) was used to estimate the reproductive outcomes of different groups based on the Kaplan-Meier method. R package (survivalROC) and time-dependent receiver operating characteristics (timeROC) curves were evaluated the reproductive outcomes of the FerSig risk model.

The decision curve analysis (DCA) curves were generated to assess the net benefits with the FerSig risk model and different clinical predictors (including age, BMI, IR, serum lipids) by using the R package (ggDCA). The DCA curves evaluated the clinical diagnostic value of the risk model and different clinical predictors.

The prognosis of PCOS was predicted using the nomogram. The live birth probabilities at 6, 28, and 37 weeks were calculated by using the R package (regplot). The nomogram calibration curves were plotted based on all proteins of the FerSig risk model and clinical predictors to observe the consistency between the predicted probability and the actual live birth. The closer its value is to 1, the better the performance.

### Immune Microenvironment Analysis

PCOS patients’ prognosis was altered by the invasion of immune cells (16, 29, 30). To investigate the relationship between ferroptosis status and immune cell infiltration in PCOS patients, we used single-sample gene set enrichment analysis (ssGSEA) and calculated the infiltration scores of 34 immune cells subpopulations using R packages (GSVA, GSEABase) (31, 32). The 34 immune cells subpopulations gene matrix transposed (gmt) file was customized by referring to these studies (**Supplementary Table S2**) (31, 33, 34). The correlation analysis between risk score and immune score were analyzed based on R packages (limma, estimate, ggExtra, ggplot2, ggpubr). Then we further analyzed the correlation expression of the FerSig risk model proteins with 9 infiltrating immune cells (B cells, T cells, NK cells, monocytes, macrophages, mast cells, dendritic cells, neutrophils, and eosinophils) and their subpopulations.

### Statistical Analysis

The SPSS software (IBM, version 25.0) was used to calculate clinical data. The proteomic data were analyzed by R software (version 4.1.1, https://www.R-project.org). The mean ± standard deviation (SD) was used to show the results data. It was decided whether the data had a normal distribution using the Kolmogorov–Smirnov test. The student t-test and the unpaired t-test were used for normal distribution data. The Mann–Whitney U test was used for non-normal distribution continuous variables for statistical comparisons. It was found that both clinical data and proteomic data were continuous variables with non-normal distribution *via* statistical tests. These data were compared by Mann–Whitney U-test. When the p-value was less than 0.05, the results were deemed statistically significant.

## Results

### Clinical and Prognosis Data Evaluation

To understand the molecular mechanism underlying the dampened endometrial receptivity of PCOS, we collected endometrium tissues of 33 PCOS and 7 controls. Age, BMI, several endocrine and biochemical markers were recorded as the clinical data. Women with PCOS and the control group had no significant differences in age (*p*=0.452). Women with PCOS significantly exhibited BMI, higher serum hormone levels (AMH, T, FSH, LH), and higher biochemical levels (Glucose, Insulin, HOMA-IR) compared with those of the control group (*p*<0.05). Conversely, women with PCOS had significantly thinner endometrium than that of the control group (*p*<0.001) (**Table 1**, **Figure 1**). The gestational duration differences between PCOS and the control group were not significant (*p*>0.05), but the pregnancy outcomes were (*p*=0.043, **Table 1**).

**Table 1:** Clinical and prognosis data of patients with PCOS and controls.

Variable	PCOS (n = 33)	Control (n = 7)	U/χ^2^ value	*p* value
**Clinical data**				
**Demographic characteristics**				
Age (years)	25.82±3.15	27.00±2.94	94.50	0.452
BMI (kg/m^2^)	24.17±3.79	21.39±2.81	55.00	0.031*
**Clinical indicators**				
AMH (ng/mL)	8.96±4.08	1.81±1.07	8.00	<0.001**
Basal FSH (IU/L)	6.82±1.65	5.67±0.83	52.00	0.024*
Basal LH (IU/L)	11.85±4.27	5.34±0.62	23.00	0.001*
LH/FSH Ratio	1.77±0.66	0.95±0.12	22.00	0.001*
Basal T (ng/dL)	42.12±17.56	24.69±11.36	45.00	0.012*
Glucose (mmol/L)	5.22±0.54	4.51±0.41	33.00	0.003*
Insulin (mIU/L)	19.51±12.72	8.97±3.90	47.50	0.015*
HOMA-IR	4.60±3.16	1.79±0.79	33.00	0.003*
ET (mm)	4.70±2.11	9.91±1.24	8.00	<0.001**
**Prognosis data**				
**Gestational duration (weeks)**				
Gestational time of live birth	38.10±2.00	37.86±1.07	53.50	0.351
Adverse gestational time	5.77±5.07	–	–	–
**Pregnancy outcomes**				
Live birth n(%)	20(60.6)	7(100)	4.085	0.043*
Adverse gestation n(%)	13(39.4)	0(0)		

All data are mean±SD values. BMI Body mass index; AMH, anti-Müllerian hormone; FSH, follicle stimulating hormone; LH, luteinizing hormone; T, testosterone; HOMA-IR, homeostasis model assessment insulin resistance; ET, endometrial thickness; U value of Mann-whitney U test, χ^2^ value of chi-square test, *, ** statistically significant.

### Endometrial Proteomic Analysis

The endometrial tissues were subjected to quantitative proteomic analysis to identify the proteins responsible for PCOS endometrial dysfunction-related. All samples had 54321 peptides and 5331 proteins identified. **Figure 1C** shows the number of proteins and peptides discovered in distinct groups of samples. A total of 4425 of 5331 proteins were overlapped in PCOS and control group, as shown in the Veen diagram, **Figure 1**.

### Identification and Enrichment of DEPs

**Figure 2:**
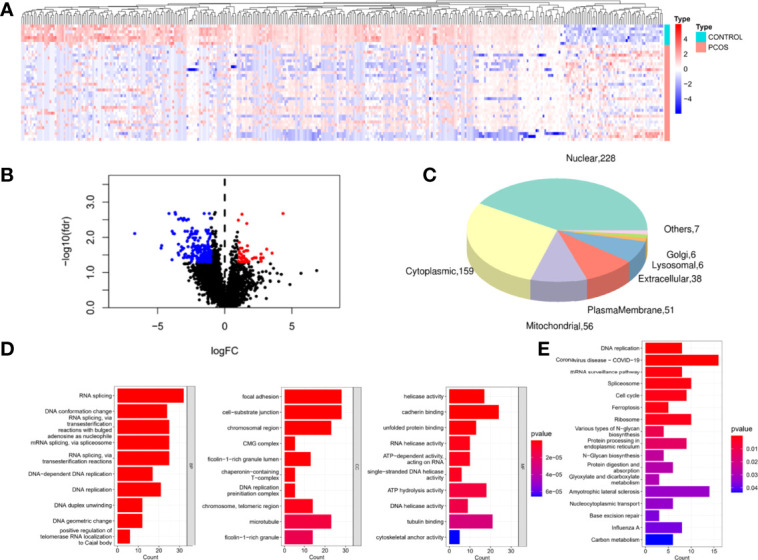
Identification and functional enrichment of DEPs.

Using hierarchical clustering analysis, we identified 71 up-regulated proteins and 320 down-regulated proteins compared with the control samples (**Figures 2A**). We discovered 492 proteins of organelles by subcellular localization prediction system analysis, 31 of which were detected in mitochondria (**Figure 2**). They were shown to be predominantly involved in cell, metabolic, and organelle functions using GO functional analysis (**Figure 2D**). GO analysis revealed that DEPs were significantly enriched in the biological processes (BP) of RNA splicing, DNA conformation change, DNA replication, conformation change, DNA replication, and DNA geometric change. DEPs in the cellular components (CC) were primarily enriched in focal adhesion, cell-substrate junction, chromosomal region, ficolin-1-rich granule, chaperonin-containing T-complex, DNA replication, and microtubule. The molecular functions (MF) were enriched in helicase activity, cadherin binding, cadherin binding, tubulin binding, and cytoskeletal anchor activity. KEGG pathway analysis also revealed 17 pathways with high enrichment of DEPs, and these pathways included DNA replication, spliceosome, cell cycle, and ferroptosis, to name a few (**Figure 2**). Cell proliferation and cell cycle were shown to be linked to the proteins.

### Ferroptosis Relative Proteins Differential Analysis

**Figure 3:**
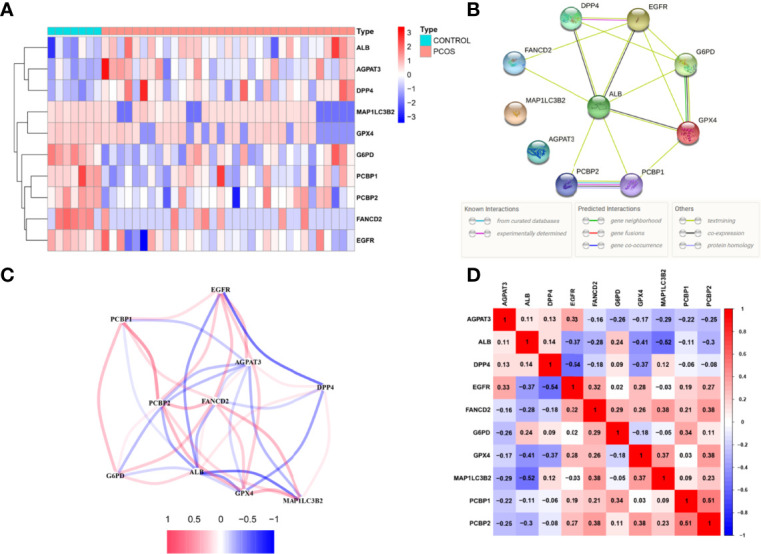
Ferroptosis relative proteins differential analysis.

Ferroptosis, which was less investigated and enriched in PCOS, was chosen as the pathway of choice in our studies. To delineate the 10 candidate ferroptosis DEPs were identified using the limma R package and machine learning, with 3 up-regulated (AGPAT3, ALB, DPP4) and 7 down-regulated proteins (MAP1LC3B2, G6PD, FANCD2, PCBP1, PCBP2, EGFR, GPX4) (**Figure 3**). G6PD, DPP4, GPX4, ALB, EGFR, FANCD2, PCBP1, and PCBP2 had an interaction score of low confidence 0.15 in the protein-protein interaction network of these potential ferroptosis DEPs (**Figure 3**). The expressions of candidate ferroptosis DEPs were used to establish a correlated network (**Figure 3**) and correlated heatmap (**Figure 3**).

### Establishment and Evaluation of the FerSig Risk Prognostic Model

**Figure 4:**
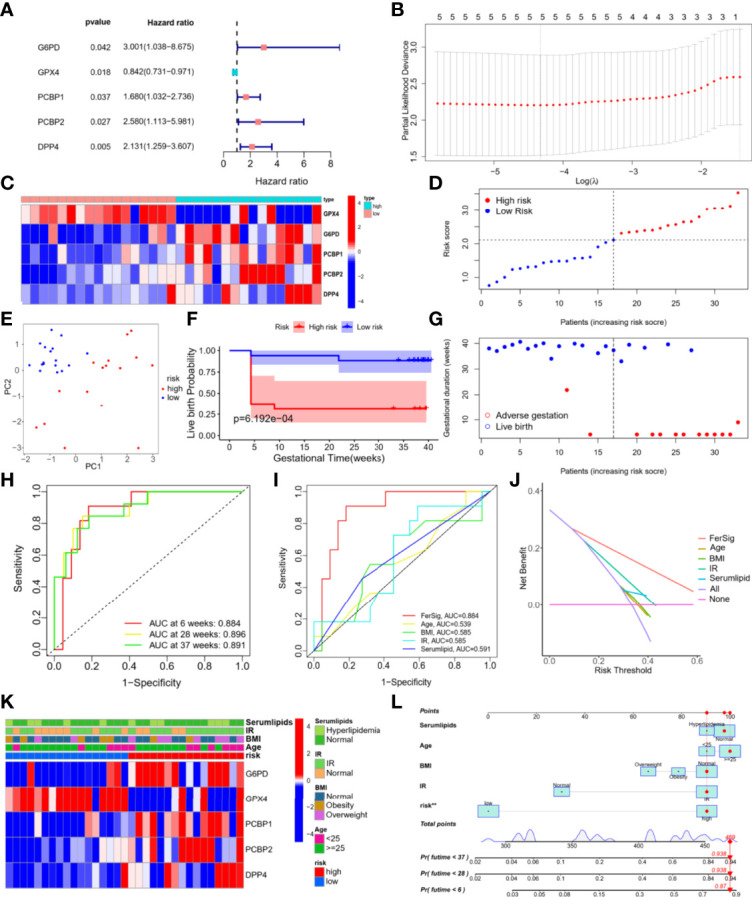
Establishment and evaluation of the FerSig risk prognostic model.

Prognostic data from 33 PCOS patients and expressions of ferroptosis DEPs were used to construct the FerSig risk prognostic model. Then univariate Cox regression analysis and Lasso regression were used to identify 5 independent prognostic ferroptosis proteins, which were utilized to establish a prognostic model. 5 independent prognostic ferroptosis proteins were G6PD, DPP4, GPX4, PCBP1, and PCBP2. (**Figure 4**). The prognostic signature was identified and performed survival analyses based on the LASSO regression (optimal value of λ) according to the optimal cut-off expression value of each candidate ferroptosis protein (**Figure 4**). The following is how the FerSig risk prognostic signature was established based on prognostic data and 5 ferroptosis proteins as follows: FerSig (PCOS)= 0.00111362055530056 × expr (G6PD) + 0.000645037118531247 × expr (DPP4) - 0.00217906042357578 × expr (GPX4) + 0.000140537487448025×expr (PCBP1) + 0.000131772720458687 × expr (PCBP2). In this FerSig score, positive coef of G6PD, PCBP1, PCBP2, and DPP4 suggested that they might be risk factors for a poor prognosis, while negative ceof of GPX4 indicated that it could be a protective factor for live birth. FerSig(PCOS) represents a prognostic risk score, and expr (proteins) represents the expression amount of the protein (**Figure 4**).

Based on their FerSig scores, PCOS patients were divided into high and low-risk groups using the median risk score (2.109) (**Figure 4**). PCA was utilized to distinguish between the two distinct categories of risk. Patients in the high-risk and low-risk groups were found to be distributed in distinct directions by the PCA results (**Figure 4**). The reproductive outcomes of the low-risk group were consistently better than those of the high-risk group, as shown by Kaplan-Meier analysis (*p*<0.01, **Figure 4**). In the high-risk group, the live birth rate was 31.3%, whereas it was 88.2% in the low-risk group. As the FerSig risk score increased, the live birth status in the high-risk group was significantly poorer than that in the low-risk group (**Figure 4**). The correlation between each phenotype of PCOS and risk score were compared, which indicates that the phenotype of our PCOS does not affect the prediction ability of the model (*p*>0.05, **Supplementary Figure 2**) (35).

The risk score for reproductive outcomes was tested using time-dependent ROC curve analysis. With an AUC of 0.893 at 6 weeks, 0.904 at 28 weeks, and 0.906 at 37 weeks, the signature of the five ferroptosis DEPs had outstanding predictive validity (**Figure 4**). The FerSig’s AUC at 6 weeks was 0.884, substantially higher than the AUCs of age (AUC=0.539), BMI (AUC=0.585), IR (AUC=0.585), and blood lipids (AUC=0.591) when compared to clinical data (**Figure 4**). These ROC prediction findings showed that FerSig had a superior prognostic performance as evidenced by these clinical data prediction comparisons. When making clinical decisions, DCA indicated that the net benefit (NB) of the FerSig risk score might be better than other clinical indexes. Although the NBs derived from clinical prognostic data (such as a patient’s age, BMI, IR, and serum lipids) were convenient, they were still inferior to those obtained by the FerSig model (**Figure 4**). The clinical features were as follows: age: <25 and ≥ 25-year-old, BMI: normal, obesity, and overweight, IR: IR and normal, and serum lipids: normal and hyperlipidemia. The distribution of clinical features in different risk groups was not balanced (**Figure 4**). FerSig proteins and an additional nomogram were built to predict the individual’s unfavorable gestational rate at 6, 28, and 37 weeks using a combination of clinical features and the FerSig risk score. For example, the overall score of 469 for a 29-year-old PCOS patient with normal serum lipids (96 points), normal BMI (91 points), insulin resistance (91 points), and a high-risk score (91 points) equates to an unfavorable gestational rate of 6 weeks (87 percent), 28 weeks (93.8 percent), and 37 weeks (93.8 percent). The FerSig risk level appears to have a greater impact on PCOS prognosis than traditional clinical features. The FerSig risk signature provides an important supplement for the adverse gestational of PCOS analysis (**Figure 4**).

### Immune Microenvironment Analysis

**Figure 5:**
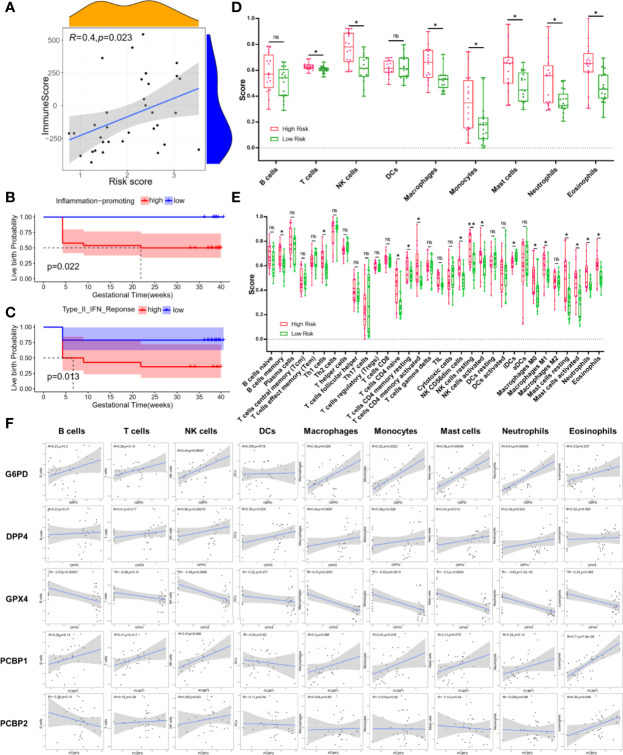
Immune microenvironment analysis.

FerSig proteins expressions and the immunological microenvironment were worthy of further investigation. The R package (“estimate”) was used to examine the correlation between the proportion of immunity and risk score in the low-risk and high-risk categories. Immune and risk scores were shown to have a moderately significant correlation (R=0.4, *p*=0.023, **Figure 5**). To further illustrate this point, the low live birth probability (all *p*<0.05, **Figures 5B**) was caused by high estimates of immune activities such as inflammation-promoting and type II IFN response. The association between immune cell infiltration and PCOS patients’ reproductive outcomes risk was also examined. T cells, NK cells, macrophages, monocytes, mast cells, neutrophils, and eosinophils exhibited greater infiltration scores in the high-risk samples than those in the low-risk samples (all *p*<0.05, **Figure 5**), according to the plot of immune cell infiltration. The results of 34 subpopulations immune cells showed that B cells memory, Th1 cells, T cells CD4 naïve, T cells CD4 memory resting, T cells CD4 memory activated, NK CD56dim cells, NK cells resting, NK cells activated, iDCs, macrophages M0, M1, mast cells resting, mast cells activated, neutrophils, and eosinophils had higher infiltration score than that in the low-risk samples (all p<0.05, **Figure 5**). The person’s correlation analysis was used to examine the connection between 5 FerSig proteins and 9 immune cells. The FerSig proteins were shown to be substantially linked with the infiltration levels of nine immune cells (**Figure 5**). G6PD, DPP4, PCBP1, and PCBP2 were significantly positive correlated with infiltration levels of most immune cells. However, GPX4 was significantly negative correlated with infiltration levels of B cells, NK cells, macrophages, monocytes, mast cells, and neutrophils.

## Discussion

Anovulation is not the sole reason for infertility in PCOS. The defective endometrium causing recurrent abortion and implantation failure can also be responsible for it (11). Therefore, ovulation dysfunction and poor endometrial receptivity are the main factors of infertility in PCOS patients (17). Studies have shown that the change of oocyte competence (OC) may be linked to the low fertility of PCOS women, however, OC varies among PCOS phenotypes and associated morbidities (36, 37). Other studies find that non-PCOS infertile individuals obtained oocyte donation from PCOS patients, and the rates of fertilization, implantation, and clinical pregnancy did not diminish, suggesting that the oocyte quality of PCOS patients might be normal (38–40). Compared with the non-PCOS population, PCOS patients who recovered ovulation with treatment had lower clinical pregnancy and live birth rates, suggesting that impaired endometrial receptivity was the one of factors leading to infertility in PCOS patients (41). While improving endometrial function in PCOS patients may not always cure infertility, it could lessen poor reproductive outcomes like as recurrent abortion and implantation failure in PCOS patients who resumed ovulation.

PCOS patients’ impaired endometrial receptivity and reproductive outcomes may be affected by changes in numerous signal pathways as the disease progresses (17, 42, 43). In our study, the DEPs were shown to be involved in ferroptosis and mitochondrial dysfunction based on the KEGG pathway and subcellular location prediction system. Ferroptosis is a cell death characterized by increased iron content, lipid peroxidation, and plasma membrane damage (26, 27). Among many functions in the human body, iron is critical to cell biological processes such as metabolism, growth, and proliferation. Balanced absorption, systemic transport, cellular uptake, and storage of iron ensure homeostasis of iron metabolism equilibrium (27, 44). Iron accumulation in a particular range may stimulate cell proliferation, while excessive iron accumulation in cells will result in excessive production of reactive oxygen species and further ferroptosis. With ferroptosis, there was no production of apoptotic bodies and autophagic vacuoles. Morphologically, the volume of mitochondria decreases, the membrane density rises, and the cristae shrink in cells with ferroptosis (26, 27). In intracellular biochemical reaction, when the metabolism of the cellular antioxidant system is abnormal, glutathione (GSH) is exhausted, the activity of glutathione peroxidase 4 (GPX4) decreases, and lipid peroxides cannot be metabolized by glutathione reduction reaction catalyzed by GPX4. Then Fe^2+^ oxidizes lipids in a manner like the Fenton reaction, produces a large number of reactive oxygen species (ROS), and promotes ferroptosis of cells. PCOS has been reported to be related to increased reactive oxygen species (ROS) and reduced expression or activity of antioxidant enzymes (19, 20, 23). Mitochondria that release ROS play a significant role in cell energy metabolism and cell death, mitochondrial dysfunction at the cellular level may have an impact on the metabolic equilibrium of PCOS patients (45–47). Oxidative stress based on increased ROS production can induce mitochondrial components impairment, such as mtDNA, proteins, and lipids (18, 21, 22). The comprehension functional mechanisms of ferroptosis have shown that ferroptosis also are crucial for features of PCOS. This study revealed that the ferroptosis pathway was implicated in PCOS patients’ endometrium.

It is still challenging to reliably predict reproductive outcomes using traditional clinical features, and there are few PCOS-specific biomarkers in PCOS patients (48, 49). As a result, more accurate prognostic models for PCOS patients are urgently needed. Therefore, we employed a computer model that incorporated the expression patterns of FerSig proteins. Then we constructed a FerSig model using 5 proteins (GPX4, DPP4, G6PD, PCBP1, PCBP2).

Previous studies have illustrated that 5 proteins are critically involved in tumors, and these proteins are rarely studied in PCOS. As one of the markers of ferroptosis, some studies have shown that GPX4 plays an important role in the occurrence of PCOS (50, 51). TP53 has been shown to inhibit the activity of dipeptidyl-peptidase-4 (DPP4), which promotes the occurrence of ferroptosis (52). DPP4 regulates the bioactivity of many peptides through cleavage and inactivation including the incretin hormones, glucagon like peptide-1 (GLP-1), and glucose-dependent insulinotropic polypeptide, and then inhibitors of DPP4 are used therapeutically to improve the blood glucose level (53, 54). AMH levels and indicators of insulin resistance are closely connected with DPP4, and DPP4 might be an additional characteristic of the metabolic imbalances associated with PCOS (55, 56). In the study of PCOS patients and animal models, it was found that the activity of glucose-6-phosphate dehydrogenase (G6PD) is inhibited by hyper androgen, thus inhibiting the pentose phosphate pathway (PPP) and reducing the biosynthesis of nicotinamide adenine dinucleotide phosphate (NADPH). Then NADPH causes the increase of ROS and PUFAs in oocytes (57, 58). Among the poly r(C) binding protein (PCBP) family members, PCBP1/2 is known as iron chaperones, which selectively bring into play Fe-GSH function *via* metal-mediated protein-protein interactions in the cytosol (59–61). PCBP1/2 directly binds ferrous iron and mediates its transfer to or from client proteins *via* metal-mediated protein-protein interactions (59). In addition, the studies have found that PCBP1/2 is downregulated, the hepatocytes were shot of the ability to manage the chemical reaction of iron, suggesting that it plays an important role in preventing ferroptosis (59). The study has revealed that the decrease of PCBP1 increases the androgen receptor (62). Therefore, we speculate that PCBPs are related to the progression of PCOS.

The 33 PCOS patients were classified into the high and low-risk groups with significantly different live birth. We further demonstrated that a high-risk score was associated with the adverse gestational rate. In different clinical features, we also found that the FerSig had different pregnancy outcomes. Time-dependent ROC curve of the signature of 5 FerSig proteins predicted PCOS patients’ pregnancy outcomes. In addition, the prognostic values based on the FeriSig were independent of other clinical variables, including age, BMI, IR, and serum lipids.

A state of chronic low-grade inflammation of PCOS might be the significant reason for decreased endometrial receptivity and adverse fertility. It is reported that sex hormones and immune cells are interactive factors in chronic low-grade inflammation of PCOS, and the inflammation may affect vital physiological processes that ultimately cause infertility (63, 64). In our study, the immune score was rising with the increase of risk score in PCOS patients. It could be reasonably assumed that ferroptosis may be critically involved in PCOS immunity, and the inflammation-promoting and type II IFN response may cause low live birth probability in PCOS. Previous studies indicated chronic low-grade inflammation is a key contributor to the pathogenesis of PCOS, and it would affect the occurrence of insulin resistance and ovarian dysfunction (64–66). Furthermore, *in vitro* studies have demonstrated the ability of inflammation-promoting stimuli to upregulate the ovarian theca cell steroidogenic enzyme responsible for androgen production (67). The type II IFN response in the endometrium of PCOS has not been reported until now.

Our study implied that PCOS patients had altered endometrial immune cells, which might indicate a state of chronic low-grade inflammation. The scores of NK cells were the most statistically different between the low-risk and the high-risk group. Previous studies have reported that NK cells (including the resting and activated NK cells) of the endometrium were closely associated with IR and low-grade chronic inflammatory state in PCOS (68). CD56dim NK cells were positively correlated with recurrent pregnancy loss and implantation failure (69–71). These studies indicate that toxic CD56dim NK cells will reduce the function of the endometrium and affect embryo implantation. T cells play a crucial role in mediating inflammation and insulin resistance and promote granular cell development and selection of the ovarian follicles, along with cytotoxic signals to induce the apoptosis of granulosa cells (63). More CD4+ T cells in the high-risk group were guided to differentiate into Th cells by the cytokine milieu. IL-12 can drive the differentiation of T cells to Th1 cells. In contrast, IL-13 drives the differentiation of T cells to Th2 cells (72, 73). IL-12 levels in the follicular fluid of PCOS patients were found to be significantly higher than those of women with regular ovulation, whereas the IL-13 levels decreased significantly, and it was suggested that Th1 type immunity was predominant in systemic immunization of PCOS patients (74, 75). Macrophages are the most abundant immune cells within the adipose. Accumulation of macrophages can also result in an influx of a plethora of proinflammatory cytokines and chemokines (IL-1, IL-6, IL-10, IL-12, and TNF-α) into the circulatory system at the same time, leading to a state of systemic chronic low-grade inflammation (76, 77). The macrophage M0 polarization translates to a pro-inflammatory M1 state, and then M1 stimulates the production of androgen and inhibits insulin sensitivity by producing TNF-α and IL-6 in PCOS (68, 78, 79). Mast cells could take part in immune regulation by a variety of cytokines. A previous study found that mast cells might participate in the pathogenesis of PCOS by releasing cytokines/chemokines and selectively inducing T cell activation (80). Mast cells were overactive in abortion patients’ endometrium by creating a pro-inflammatory microenvironment, suggesting that overexpression of mast cells in the endometrium may be one of the reasons for poor fertility in patients with PCOS (81). Neutrophils could infiltrate tissue to clean up dysfunctional and dead cells. With PCOS hyperandrogen, the neutrophils count increased along, which resulted in a state of chronic low-grade inflammation (82).

There are still some limitations that require further study. This study may only provide individual treatment direction for PCOS patients, who were nonovarian factors combined with infertility and poor reproductive outcomes. Although FerSig has been established in the PCOS DIA data set, it required more independent data sets to verify the FerSig to guarantee its reliability and replicability. Though functional analysis has revealed the correlation between the ferroptosis-related proteins signature and immune-related biological processes, the regulatory mechanisms of the decreased endometrial receptivity in PCOS patients are needed to understand *via* large numbers of verification experiments.

## Conclusion

A FerSig model based on 5 ferroptosis-associated proteins has been constructed in this work. Ferroptosis-associated proteins may have a role in determining the reproductive outcomes of PCOS patients with decreased endometrial receptivity. We also found a strong correlation between the prognosis of PCOS and the endometrial immunological microenvironment connected to FerSig proteins, indicating that immunotherapy may have an impact on PCOS. Using the FerSig as a starting point and resource for additional research into treatment options is crucial. We expect the FerSig model to provide the theoretical basis for further research into the function and resource of ferroptosis in the endometrium, as well as a key approach to customizing individual treatment decision-making.

## Data Availability Statement

The datasets presented in this study can be found in online repositories. The names of the repository/repositories and accession number(s) can be found below: The mass spectrometry proteomics data have been deposited to the ProteomeXchange Consortium (http://proteomecentral.proteomexchange.org) via the iProX partner repository with the dataset identifier PXD032383.

## Ethics Statement

The studies involving human participants were reviewed and approved by the Ethics Committee of Lanzhou University Second Hospital. The patients/participants provided their written informed consent to participate in this study.

## Author Contributions

The study conception and design were performed by JZ and FW. Material preparation, data collection, and analysis were performed by JZ, ND, WX, and XY. The first draft of the manuscript was written by JZ and ND. The illustration is drawn by ZJ. All authors commented on previous versions of the manuscript. All authors read and approved the final manuscript.

## Funding

This work was funded by Science Foundation of Lanzhou University (Grant No. 054000229) and the Science Foundation of Lanzhou University Second Hospital (Grant No. CY2018-MS12).

## Conflict of Interest

The authors declare that the research was conducted in the absence of any commercial or financial relationships that could be construed as a potential conflict of interest.

## Publisher’s Note

All claims expressed in this article are solely those of the authors and do not necessarily represent those of their affiliated organizations, or those of the publisher, the editors and the reviewers. Any product that may be evaluated in this article, or claim that may be made by its manufacturer, is not guaranteed or endorsed by the publisher.
