# Supplementary figures and images for: Quantitative Proteomics Reveals That a Prognostic Signature of the Endometrium of the Polycystic Ovary Syndrome Women Based on Ferroptosis Proteins
Source: Front Endocrinol (Lausanne). 2022 Jul 14;13:871945. doi: 10.3389/fendo.2022.871945 (PMC9330063; doi:10.3389/fendo.2022.871945)

**Supplementary Figure 1 Coomassie blue staining of 40 samples**

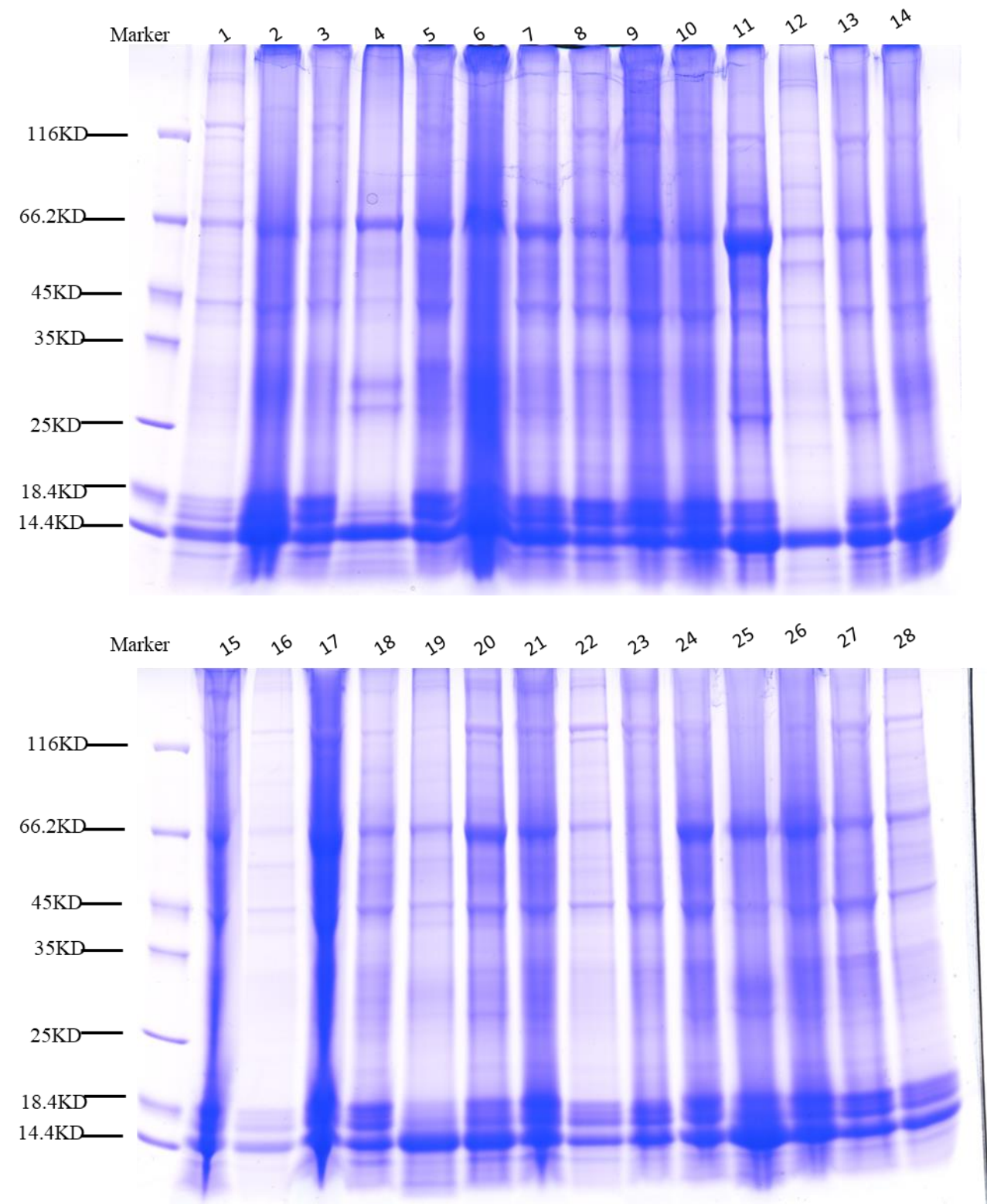

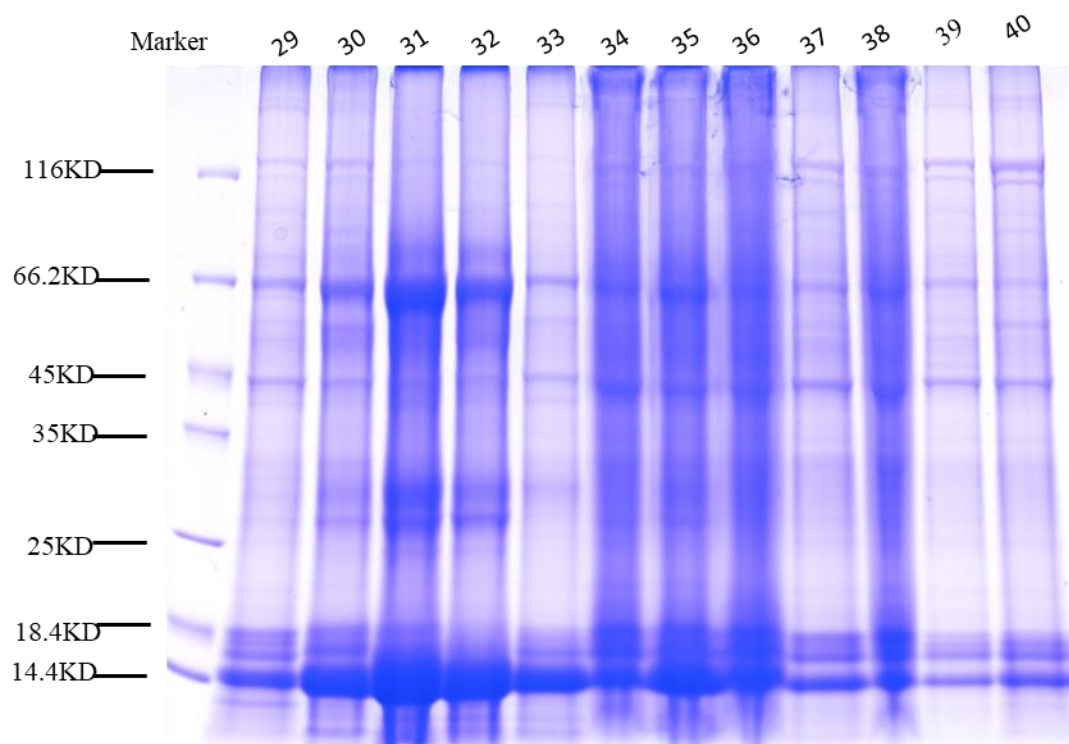

Supplement: Supplementary file 1 [file Image_1.pdf]
