# Supplementary material for: Quantitative Proteomics Reveals That a Prognostic Signature of the Endometrium of the Polycystic Ovary Syndrome Women Based on Ferroptosis Proteins
Source: Front Endocrinol (Lausanne). 2022 Jul 14;13:871945. doi: 10.3389/fendo.2022.871945 (PMC9330063; doi:10.3389/fendo.2022.871945)
Supplement: Supplementary file 2 [file Image_2.pdf]

**Supplementary Figure 2**

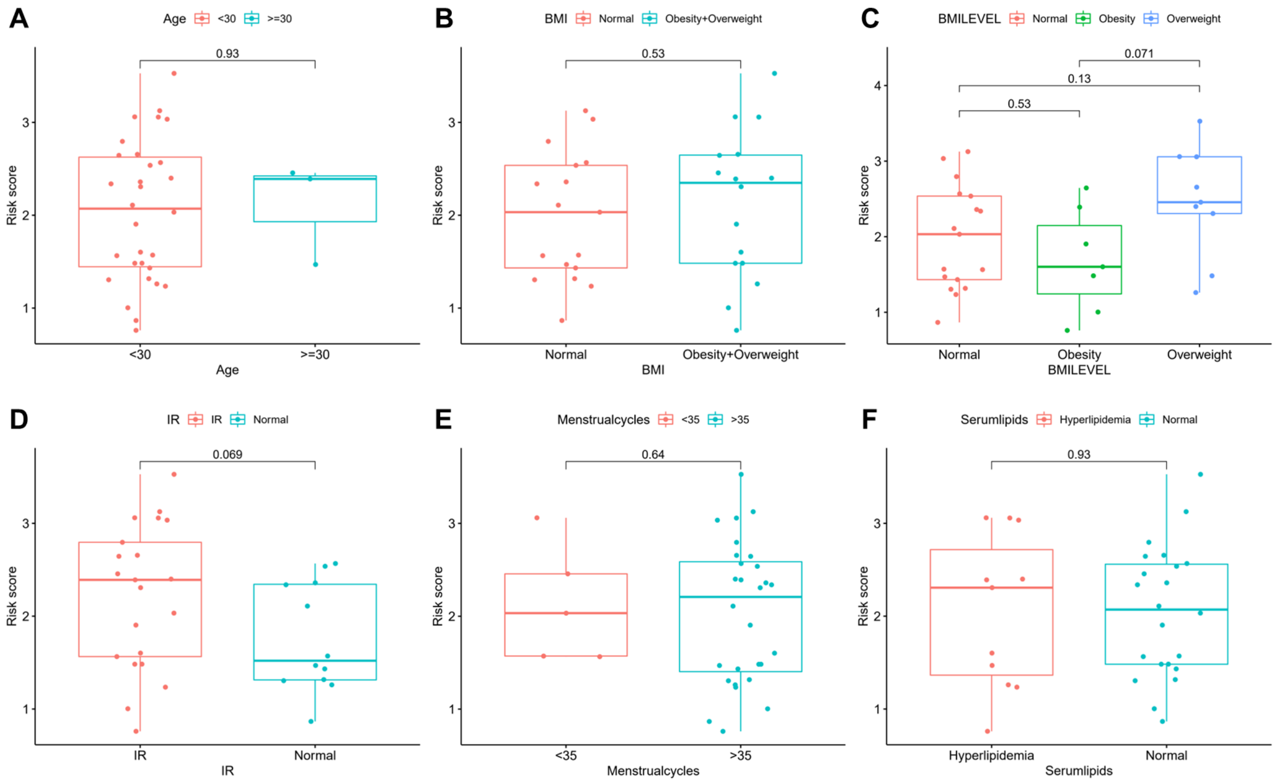


**Supplementary Figure 2 Correlation analysis between PCOS phenotype and risk score**

**A.** The correlation plot of age (≥30 and <30) and risk scores ($p$=0.93). **B.** The correlation plot of BMI (normal and obesity + overweight) and risk scores ($p$=0.53). **C.** The correlation plot of BMI (normal, obesity, and overweight) and risk scores (all $p$>0.05). **D.** The correlation plot of insulin resistance (IR and normal) and risk scores ($p$=0.069). **E.** The correlation plot of menstrual cycles (≥35 and <35) and risk scores ($p$=0.64). **F.** The correlation plot of serum lipids (hyperlipidemia and normal) and risk scores ($p$=0.93).
