# Supplementary material for: Quantitative Proteomics Reveals That a Prognostic Signature of the Endometrium of the Polycystic Ovary Syndrome Women Based on Ferroptosis Proteins
Source: Front Endocrinol (Lausanne). 2022 Jul 14;13:871945. doi: 10.3389/fendo.2022.871945 (PMC9330063; doi:10.3389/fendo.2022.871945)
Supplement: Supplementary file 3 [file Table_1.docx]

**Supplementary Tables Legends**

**Supplementary Table S1 Ferroptosis protein-coding genes**

**Supplementary Table S2 The 34 immune cells gene matrix transposed (gmt) file**

**Supplementary Table S1 Ferroptosis protein-coding genes**

| **No** | **Gene Symbol** | **Description** |
| --- | --- | --- |
| 1 | ABCC1 | ATP binding cassette subfamily C member 1 |
| 2 | ACO1 | aconitase 1 |
| 3 | ACSF2 | acyl-CoA synthetase family member 2 |
| 4 | ACSL3 | acyl-CoA synthetase long chain family member 3 |
| 5 | ACSL4 | acyl-CoA synthetase long chain family member 4 |
| 6 | ACVR1B | activin A receptor type 1B |
| 7 | AGPAT3 | 1-acylglycerol-3-phosphate O-acyltransferase 3 |
| 8 | AIFM2 | apoptosis inducing factor mitochondria associated 2 |
| 9 | AKR1C1 | aldo-keto reductase family 1 member C1 |
| 10 | AKR1C2 | aldo-keto reductase family 1 member C2 |
| 11 | AKR1C3 | aldo-keto reductase family 1 member C3 |
| 12 | ALB | albumin |
| 13 | ALOX12 | arachidonate 12-lipoxygenase, 12S type |
| 14 | ALOX12B | arachidonate 12-lipoxygenase, 12R type |
| 15 | ALOX15 | arachidonate 15-lipoxygenase |
| 16 | ALOX15B | arachidonate 15-lipoxygenase type B |
| 17 | ALOX5 | arachidonate 5-lipoxygenase |
| 18 | ALOXE3 | arachidonate lipoxygenase 3 |
| 19 | ANGPTL7 | angiopoietin like 7 |
| 20 | ANO6 | anoctamin 6 |
| 21 | ARNTL | aryl hydrocarbon receptor nuclear translocator like |
| 22 | ARRDC3 | arrestin domain containing 3 |
| 23 | ASNS | asparagine synthetase (glutamine-hydrolyzing) |
| 24 | ATF3 | activating transcription factor 3 |
| 25 | ATF4 | activating transcription factor 4 |
| 26 | ATG13 | autophagy related 13 |
| 27 | ATG16L1 | autophagy related 16 like 1 |
| 28 | ATG3 | autophagy related 3 |
| 29 | ATG4D | autophagy related 4D cysteine peptidase |
| 30 | ATG5 | autophagy related 5 |
| 31 | ATG7 | autophagy related 7 |
| 32 | ATM | ATM serine/threonine kinase |
| 33 | ATP5MC3 | ATP synthase membrane subunit c locus 3 |
| 34 | ATP6V1G2 | ATPase H+ transporting V1 subunit G2 |
| 35 | AURKA | aurora kinase A |
| 36 | BACH1 | BTB domain and CNC homolog 1 |
| 37 | BAP1 | BRCA1 associated protein 1 |
| 38 | BECN1 | beclin 1 |
| 39 | BID | BH3 interacting domain death agonist |
| 40 | BLOC1S5-TXNDC5 | BLOC1S5-TXNDC5 readthrough (NMD candidate) |
| 41 | BNIP3 | BCL2 interacting protein 3 |
| 42 | BRD4 | bromodomain containing 4 |
| 43 | CA9 | carbonic anhydrase 9 |
| 44 | CAPG | capping actin protein, gelsolin like |
| 45 | CARS1 | cysteinyl-tRNA synthetase 1 |
| 46 | CAV1 | caveolin 1 |
| 47 | CBS | cystathionine beta-synthase |
| 48 | CD44 | CD44 molecule (Indian blood group) |
| 49 | CDKN1A | cyclin dependent kinase inhibitor 1A |
| 50 | CDKN2A | cyclin dependent kinase inhibitor 2A |
| 51 | CDO1 | cysteine dioxygenase type 1 |
| 52 | CEBPG | CCAAT enhancer binding protein gamma |
| 53 | CHAC1 | ChaC glutathione specific gamma-glutamylcyclotransferase 1 |
| 54 | CHMP5 | charged multivesicular body protein 5 |
| 55 | CHMP6 | charged multivesicular body protein 6 |
| 56 | CISD1 | CDGSH iron sulfur domain 1 |
| 57 | CISD2 | CDGSH iron sulfur domain 2 |
| 58 | CS | citrate synthase |
| 59 | CXCL2 | C-X-C motif chemokine ligand 2 |
| 60 | CYBB | cytochrome b-245 beta chain |
| 61 | DDIT3 | DNA damage inducible transcript 3 |
| 62 | DDIT4 | DNA damage inducible transcript 4 |
| 63 | DNAJB6 | DnaJ heat shock protein family (Hsp40) member B6 |
| 64 | DPP4 | dipeptidyl peptidase 4 |
| 65 | DRD4 | dopamine receptor D4 |
| 66 | DRD5 | dopamine receptor D5 |
| 67 | DUOX1 | dual oxidase 1 |
| 68 | DUOX2 | dual oxidase 2 |
| 69 | DUSP1 | dual specificity phosphatase 1 |
| 70 | EGFR | epidermal growth factor receptor |
| 71 | EGLN2 | egl-9 family hypoxia inducible factor 2 |
| 72 | EIF2AK4 | eukaryotic translation initiation factor 2 alpha kinase 4 |
| 73 | EIF2S1 | eukaryotic translation initiation factor 2 subunit alpha |
| 74 | ELAVL1 | ELAV like RNA binding protein 1 |
| 75 | EMC2 | ER membrane protein complex subunit 2 |
| 76 | ENPP2 | ectonucleotide pyrophosphatase/phosphodiesterase 2 |
| 77 | EPAS1 | endothelial PAS domain protein 1 |
| 78 | FADS2 | fatty acid desaturase 2 |
| 79 | FANCD2 | FA complementation group D2 |
| 80 | FBXW7 | F-box and WD repeat domain containing 7 |
| 81 | FTHL17 | ferritin heavy chain like 17 |
| 82 | FH | fumarate hydratase |
| 83 | FLT3 | fms related receptor tyrosine kinase 3 |
| 84 | FTH1 | ferritin heavy chain 1 |
| 85 | FTL | ferritin light chain |
| 86 | FTMT | ferritin mitochondrial |
| 87 | G6PD | glucose-6-phosphate dehydrogenase |
| 88 | G6PD | glucose-6-phosphate dehydrogenase |
| 89 | GABARAPL1 | GABA type A receptor associated protein like 1 |
| 90 | GABARAPL2 | GABA type A receptor associated protein like 2 |
| 91 | GABPB1 | GA binding protein transcription factor subunit beta 1 |
| 92 | GCH1 | GTP cyclohydrolase 1 |
| 93 | GCLC | glutamate-cysteine ligase catalytic subunit |
| 94 | GDF15 | growth differentiation factor 15 |
| 95 | GLS2 | glutaminase 2 |
| 96 | GLUT13 | glucose transporters 13 |
| 97 | GOT1 | glutamic-oxaloacetic transaminase 1 |
| 98 | GPT2 | glutamic--pyruvic transaminase 2 |
| 99 | GPX2 | glutathione peroxidase 2 |
| 100 | GPX4 | glutathione peroxidase 4 |
| 101 | HAMP | hepcidin antimicrobial peptide |
| 102 | HBA1 | hemoglobin subunit alpha 1 |
| 103 | HELLS | helicase, lymphoid specific |
| 104 | HERPUD1 | homocysteine inducible ER protein with ubiquitin like domain 1 |
| 105 | HIC1 | HIC ZBTB transcriptional repressor 1 |
| 106 | HIF1A | hypoxia inducible factor 1 subunit alpha |
| 107 | HILPDA | hypoxia inducible lipid droplet associated |
| 108 | HMGB1 | high mobility group box 1 |
| 109 | HMOX1 | heme oxygenase 1 |
| 110 | HNF4A | hepatocyte nuclear factor 4 alpha |
| 111 | HRAS | HRas proto-oncogene, GTPase |
| 112 | HSD17B11 | hydroxysteroid 17-beta dehydrogenase 11 |
| 113 | HSF1 | heat shock transcription factor 1 |
| 114 | HSPA5 | heat shock protein family A (Hsp70) member 5 |
| 115 | HSPB1 | heat shock protein family B (small) member 1 |
| 116 | IDH1 | isocitrate dehydrogenase (NADP(+)) 1 |
| 117 | IFNG | interferon gamma |
| 118 | IL33 | interleukin 33 |
| 119 | IL6 | interleukin 6 |
| 120 | IREB2 | iron responsive element binding protein 2 |
| 121 | ISCU | iron-sulfur cluster assembly enzyme |
| 122 | JDP2 | Jun dimerization protein 2 |
| 123 | JUN | Jun proto-oncogene, AP-1 transcription factor subunit |
| 124 | KEAP1 | kelch like ECH associated protein 1 |
| 125 | HAVCR1 | hepatitis A virus cellular receptor 1 |
| 126 | KLHL24 | kelch like family member 24 |
| 127 | KRAS | KRAS proto-oncogene, GTPase |
| 128 | LAMP2 | lysosomal associated membrane protein 2 |
| 129 | LINC00336 | long intergenic non-protein coding RNA 336 |
| 130 | LINC00472 | long intergenic non-protein coding RNA 472 |
| 131 | LINC02806 | long intergenic non-protein coding RNA 2806 |
| 132 | LOC390705 | protein phosphatase 2 regulatory subunit B'', beta pseudogene |
| 133 | LONP1 | lon peptidase 1, mitochondrial |
| 134 | LPCAT3 | lysophosphatidylcholine acyltransferase 3 |
| 135 | LPIN1 | lipin 1 |
| 136 | LURAP1L | leucine rich adaptor protein 1 like |
| 137 | MAFG | MAF bZIP transcription factor G |
| 138 | MAP1LC3A | microtubule associated protein 1 light chain 3 alpha |
| 139 | MAP3K5 | mitogen-activated protein kinase kinase kinase 5 |
| 140 | MAPK1 | mitogen-activated protein kinase 1 |
| 141 | MAPK14 | mitogen-activated protein kinase 14 |
| 142 | MAPK3 | mitogen-activated protein kinase 3 |
| 143 | MAPK8 | mitogen-activated protein kinase 8 |
| 144 | MAPK9 | mitogen-activated protein kinase 9 |
| 145 | MIOX | myo-inositol oxygenase |
| 146 | MIR137 | microRNA 137 |
| 147 | MIR17 | microRNA 17 |
| 148 | MIR212 | microRNA 212 |
| 149 | MIR30B | microRNA 30b |
| 150 | MIR4715 | microRNA 4715 |
| 151 | MIR6852 | microRNA 6852 |
| 152 | MIR9-1 | microRNA 9-1 |
| 153 | MIR9-2 | microRNA 9-2 |
| 154 | MIR9-3 | microRNA 9-3 |
| 155 | MT1G | metallothionein 1G |
| 156 | MT3 | metallothionein 3 |
| 157 | MTDH | metadherin |
| 158 | MTOR | mechanistic target of rapamycin kinase |
| 159 | MUC1 | mucin 1, cell surface associated |
| 160 | MYB | MYB proto-oncogene, transcription factor |
| 161 | NCF2 | neutrophil cytosolic factor 2 |
| 162 | NCOA4 | nuclear receptor coactivator 4 |
| 163 | NF2 | NF2, moesin-ezrin-radixin like (MERLIN) tumor suppressor |
| 164 | NFE2L2 | NFE2 like bZIP transcription factor 2 |
| 165 | NFS1 | NFS1 cysteine desulfurase |
| 166 | NGB | neuroglobin |
| 167 | NNMT | nicotinamide N-methyltransferase |
| 168 | NOS2 | nitric oxide synthase 2 |
| 169 | NOX1 | NADPH oxidase 1 |
| 170 | NOX3 | NADPH oxidase 3 |
| 171 | NOX4 | NADPH oxidase 4 |
| 172 | NOX5 | NADPH oxidase 5 |
| 173 | NQO1 | NAD(P)H quinone dehydrogenase 1 |
| 174 | NRAS | NRAS proto-oncogene, GTPase |
| 175 | OTUB1 | OTU deubiquitinase, ubiquitin aldehyde binding 1 |
| 176 | OXSR1 | oxidative stress responsive kinase 1 |
| 177 | PANX1 | pannexin 1 |
| 178 | PCK2 | phosphoenolpyruvate carboxykinase 2, mitochondrial |
| 179 | PEBP1 | phosphatidylethanolamine binding protein 1 |
| 180 | PGD | phosphogluconate dehydrogenase |
| 181 | PHKG2 | phosphorylase kinase catalytic subunit gamma 2 |
| 182 | PIK3CA | phosphatidylinositol-4,5-bisphosphate 3-kinase catalytic subunit alpha |
| 183 | PLIN2 | perilipin 2 |
| 184 | PLIN4 | perilipin 4 |
| 185 | PML | PML nuclear body scaffold |
| 186 | PRDX1 | peroxiredoxin 1 |
| 187 | PRDX6 | peroxiredoxin 6 |
| 188 | PRKAA1 | protein kinase AMP-activated catalytic subunit alpha 1 |
| 189 | PRKAA2 | protein kinase AMP-activated catalytic subunit alpha 2 |
| 190 | PROM2 | prominin 2 |
| 191 | PSAT1 | phosphoserine aminotransferase 1 |
| 192 | PTGS2 | prostaglandin-endoperoxide synthase 2 |
| 193 | RB1 | RB transcriptional corepressor 1 |
| 194 | RELA | RELA proto-oncogene, NF-kB subunit |
| 195 | RGS4 | regulator of G protein signaling 4 |
| 196 | RIPK1 | receptor interacting serine/threonine kinase 1 |
| 197 | RPL8 | ribosomal protein L8 |
| 198 | RRM2 | ribonucleotide reductase regulatory subunit M2 |
| 199 | SAT1 | spermidine/spermine N1-acetyltransferase 1 |
| 200 | SCD | stearoyl-CoA desaturase |
| 201 | SCP2 | sterol carrier protein 2 |
| 202 | SELENOS | selenoprotein S |
| 203 | SESN2 | sestrin 2 |
| 204 | SETD1B | SET domain containing 1B, histone lysine methyltransferase |
| 205 | SIRT1 | sirtuin 1 |
| 206 | SLC1A4 | solute carrier family 1 member 4 |
| 207 | SLC1A5 | solute carrier family 1 member 5 |
| 208 | SLC2A1 | solute carrier family 2 member 1 |
| 209 | SLC2A12 | solute carrier family 2 member 12 |
| 210 | SLC2A14 | solute carrier family 2 member 14 |
| 211 | SLC2A3 | solute carrier family 2 member 3 |
| 212 | SLC2A6 | solute carrier family 2 member 6 |
| 213 | SLC2A8 | solute carrier family 2 member 8 |
| 214 | SLC38A1 | solute carrier family 38 member 1 |
| 215 | SLC3A2 | solute carrier family 3 member 2 |
| 216 | SLC40A1 | solute carrier family 40 member 1 |
| 217 | SLC7A11 | solute carrier family 7 member 11 |
| 218 | SLC7A5 | solute carrier family 7 member 5 |
| 219 | SNORA16A | small nucleolar RNA, H/ACA box 16A |
| 220 | SNX4 | sorting nexin 4 |
| 221 | SOCS1 | suppressor of cytokine signaling 1 |
| 222 | SP1 | Sp1 transcription factor |
| 223 | SQSTM1 | sequestosome 1 |
| 224 | SRC | SRC proto-oncogene, non-receptor tyrosine kinase |
| 225 | SRXN1 | sulfiredoxin 1 |
| 226 | STAT3 | signal transducer and activator of transcription 3 |
| 227 | STEAP3 | STEAP3 metalloreductase |
| 228 | STMN1 | stathmin 1 |
| 229 | TAFAZZIN | tafazzin, phospholipid-lysophospholipid transacylase |
| 230 | TF | transferrin |
| 231 | TFAP2C | transcription factor AP-2 gamma |
| 232 | TFR2 | transferrin receptor 2 |
| 233 | TFRC | transferrin receptor |
| 234 | TGFBR1 | transforming growth factor beta receptor 1 |
| 235 | TLR4 | toll like receptor 4 |
| 236 | TMBIM4 | transmembrane BAX inhibitor motif containing 4 |
| 237 | TNFAIP3 | TNF alpha induced protein 3 |
| 238 | TP53 | tumor protein p53 |
| 239 | TP63 | tumor protein p63 |
| 240 | TRIB3 | tribbles pseudo kinase 3 |
| 241 | TSC22D3 | TSC22 domain family member 3 |
| 242 | TUBE1 | tubulin epsilon 1 |
| 243 | TXNIP | thioredoxin interacting protein |
| 244 | TXNRD1 | thioredoxin reductase 1 |
| 245 | UBC | ubiquitin C |
| 246 | ULK1 | unc-51 like autophagy activating kinase 1 |
| 247 | ULK2 | unc-51 like autophagy activating kinase 2 |
| 248 | VDAC2 | voltage dependent anion channel 2 |
| 249 | VEGFA | vascular endothelial growth factor A |
| 250 | VLDLR | very low-density lipoprotein receptor |
| 251 | WIPI1 | WD repeat domain, phosphoinositide interacting 1 |
| 252 | WIPI2 | WD repeat domain, phosphoinositide interacting 2 |
| 253 | XBP1 | X-box binding protein 1 |
| 254 | YWHAE | tyrosine 3-monooxygenase/tryptophan 5-monooxygenase activation protein epsilon |
| 255 | YY1AP1 | YY1 associated protein 1 |
| 256 | ZEB1 | zinc finger E-box binding homeobox 1 |
| 257 | ZFP36 | ZFP36 ring finger protein |
| 258 | ZFP69B | ZFP69 zinc finger protein B |
| 259 | ZNF419 | zinc finger protein 419 |

**Supplementary Table S2 The 34 immune cells gene matrix transposed (gmt) file**

| **Immune Cells** | **Symbol genes** |
| --- | --- |
| B cells naive | CD72, LINC00921, LTB, GUSBP11, RASGRP2, STAP1, LY86, CD79A, MEP1A, IGHD, ZNF286A, BEND5, SPIB, FRK, FCER2, MICAL3, PSG2, MS4A1, CD1C, BLK, IGKC, UGT1A8, EAF2, NIPSNAP3B, CD180, NMBR, SELL, CD79B, FCGR2B, CD19, BACH2, IGLL3P, P2RX5, CD37, RALGPS2, BRAF, CXCR5, FCRL2, TCL1A, HLA-DOB, BCL7A, HHEX, IGHM, PNOC, CR2, PTPRCAP, GPR18, SIK1, ABCB4, SLC12A1, VPREB3, CD22, BANK1, IRF8, P2RY14, IL4R, KIAA0226L, FAIM3, ADAM28, CD69 |
| B cells memory | CXCR5, RALGPS2, RASGRP2, IGLL3P, GNG7, SIT1, SIK1, FAIM3, SLC12A1, LTB, TNFRSF17, CD19, NPIPB15, IGHM, HHEX, SPIB, CLCA3P, ZBTB32, STAP1, CD72, CD1C, MS4A1, TMEM156, GPR18, VPREB3, CD37, AIM2, IFNA10, FCGR2B, HLA-DOB, CD69, BANK1, NMBR, ALOX5, CD79B, FAM65B, CD22, FRK, IGHD, SP140, KIAA0226L, CD180, IRF8, LY86, CD27, FCRL2, DENND5B, IGKC, IL7, ADAM28, MBL2, TNFRSF13B, TRAF4, CD79A, BLK, PNOC, CCR6, P2RX5, PTPRCAP, GUSBP11, BACH2, CR2 |
| Plasma cells | TMEM156, MZB1, PAX7, HIST1H2BG, PDK1, TNFRSF17, RASGRP3, KCNG2, IGHM, GPR25, C11orf80, REN, ZBP1, RGS13, SIK1, IGKC, LOC100130100, ANGPT4, RPL3P7, P2RX5, SPAG4, EAF2, CCR10, PNOC, GUSBP11, MAN1A1, ST6GALNAC4, DENND5B, IGLL3P, AMPD1, CD38, KCNA3, IGHD, IGHE, LIME1, UGT2B17, ABCB9, ZNF165, HIST1H2AE, CD79A, CD27, HLA-DOB, MAST1, TGM5, MANEA, MROH7, ATXN8OS, FCRL2, GNG7 |
| T cells central memory  (Tcm) | LOC202134, SLC7A6, FYB, POLR2J2, MAP3K1, TIMM8A, PDXDC2, KLF12, TRAF3IP3, STX16, PCM1, ST3GAL1, DOCK9, RPP38, CASP8, ATF7IP, CYLD, MLL, USP9Y, CYorf15B, PHC3, INPP4B, PSPC1, SNRPN, CG030, FOXP1, AQP3, CLUAP1, REPS1, NEFL, CDC14A, RP11-74E24.2, PCNX, CEP68, CREBZF, TXK, HNRPH1, ATM, NFATC3 |
| T cells effect memory  (Tem) | LTK, C7orf54, EWSR1, FLI1, DDX17, AKT3, VIL2, CCR2, TBC1D5, MEFV, TRA, TBCD, GDPD5, PRKY, NFATC4 |
| Th1 cells | DUSP5, CD38, SGCB, LRP8, LTA, IL12RB2, APOD, CMAH, SYNGR3, CSF2, CD70, ZBTB32, STAT4, GGT1, IL22, ATP9A, APBB2, TBX21, EGFL6, CCL4, BTG3, DGKI, HBEGF, DPP4, DOK5, IFNG, CTLA4, LRRN3, BST2 |
| Th2 cells | AI582773, GATA3, CDC25C, LAIR2, IL26, HELLS, BIRC5, SMAD2, MB, GSTA4, SNRPD1, DHFR, PHEX, PTGSIS, ANK1, NEIL3, MICAL2, SLC39A14, EVI5, CDC7, CXCR6, LIMA1, CENPF, PMCH, WDHD1, AHI1, ADCY1 |
| T helper cells | UBE2L3, ANP32B, NUP107, CD28, FRYL, SEC24C, SLC25A12, BATF, DDX50, GOLGA8A, ASF1A, ITM2A, TRA, NAP1L4, PPP2R5C, CD4, PHF10, C13ORF34, ICOS, FUSIP1, FAM111A, ATF2, LRBA, RPA1 |
| T cells follicular helper  (Tfh) | ST8SIA1, THADA, MAP9, LEF1, MAGEH1, C18orf1, CA8, TRAC, LAG3, B3GAT1, HEY1, PASK, SH3TC1, TNFRSF4, BCL11B, CTLA4, PDCD1, LAT, CHI3L2, HIST1H4K, ICOS, RPL3P7, STK39, KIAA1324, CDK5R1, TRIB2, TRAV9-2, GPR19, SIRPG, SMAD1, ICA1, ZAP70, CD2, MAP4K1, CD247, TRAT1, TOX, MKL2, BLR1, SLC7A10, SIK1, MYO7A, CD3D, SH2D1A, TSHR, PTPN13, CXCL13, MAF, FAIM3, ATHL1, KLRB1, ZNF764, CD7, LCK, KCNK5, UBASH3A, CD40LG, LTA, CD27, RPVALB, ITK, DGKA, GZMM, CHGB, TCF7, TRAV13-1, MYO6, CD3G, TRBC1, CD69, PTPRCAP, TRAV8-6, PVRIG, CXCR5, POMT1, FOSB, FZD3, IL21, RGS1, ZBTB10 |
| Th17 cells | RORC, IL17A, IL17RA |
| T cells regulatory  (Tregs) | MBL2, LCK, TYR, LILRA4, PLCH2, CASP1, UBASH3A, NTN3, THADA, LY75, BCL2L1, ITK, JAK1, IKZF2, NPTN, HSDL2, MAP4K1, SSTR3, CHRNA6, SKAP1, METTL7A, IL1R1, CD70, TFRC, HIC1, PTPRJ, PTPRG, DPP4, GPR19, LAIR2, SIRPG, SOCS2, FRMD8, SLC35F2, GRSF1, NAB1, PTGIR, SSH1, CSF2RB, RNF145, CD28, TRAC, LTB, LAX1, ENTPD1, CD177, SIT1, CCR8, DGKA, CD2, LOC126987, TMEM184C, SPOCK2, TRAT1, ADAT2, NDFIP2, RYR1, VDR, HTATIP2, IL21R, RCAN3, IL2RB, CHST2, GPR171, NPAS1, BCL11B, TMPRSS6, ADPRH, ZBTB38, LIMA1, LAYN, SEC31B, NETO2, IL2RA, CEMP1, TIGIT, SSX1, AHCYL1, CSF1, ACSL4, CD4, CADM1, CTLA4, CD3G, LAPTM4B, FOXP3, 5-Sep, RRAGB, BATF, CD5, TRAF3, FKBP1A, HS3ST3B1, ETV7, LEPROT, GCNT1, HMGB3P30, TNFRSF18, KSR1, PCDHA5, NFE2L3, CHST7, ICOS, ACP5, CTSC, NFAT5, ZNF282, ANKRD10, CD3E, ERI1, MAGEH1, CD247, CD96, TNFRSF4, CD3D, SH2D1A, GPR1, CD27, BARX2, CD274, GZMM, LTA, CLEC2D, EFNA5, TRBC1, ZAP70, IL12RB2, IL1R2, TRAV9-2, CD6, KIRREL, PMCH |
| T cells CD8 | ZFPROTL1, IL7R, TRDC, PPP1R2, KLRC4, FLT3LG, AES, TMC6, LEF1, CD2, CD6, KLF9, PIK3IP1, PRF1, CDKN2AIP, CD96, DPP4, CD3G, KLRF1, LY9, ZNF91, C12orf47, IGKC, PTPRCAP, CST7, CD27, SFRS7, DNAJB1, RASA3, KLRB1, TCF7, TBCC, CD8A, GNLY, UBASH3A, ZEB1, NCR3, GZMB, LIME1, KLRC3, RPL3P7, CD247, GADD45A, THUMPD1, TRAV13-1, SLC16A7, PVRIG, LAG3, PF4, RBM3, CD69, C4orf15, CCL5, ARHGAP8, SH2D1A, MYST3, VAMP2, CD7, TRAV12-2, CTSW, KLRD1, GZMM, SF1, TSC22D3, LTB, ZFP36L2, BCL11B, ZNF22, CD3D, CD3E, CD8B, CRTAM, APBA2, DUSP2, ICOS, DSC1, PRR5, GPR171, GRAP2, C19orf6, TRAC, FAIM3, GZMA, GZMH, TRAT1, ABT1, GZMK, ITK, TRBC1, KLRK1, ZAP70, CAMLG, LCK, ZNF609, MAP4K1, MAP9, LEPROTL1, NKG7, PTGDR, SIRPG |
| T cells CD4 naive | GRAP2, ATHL1, CCR7, TCF7, ZNF204P, CD40LG, GZMM, CXorf57, TRAV13-1, BCL11B, SH2D1A, LY9, EPHA1, SERGEF, FLJ13197, CD7, CD2, UBASH3A, LCK, GAL3ST4, CD3D, GALR1, ZNF324, ICOS, MAP4K1, CD3G, RPL3P7, ZAP70, GPR1, DSC1, WNT7A, CD247, ITK, MAP9, TRAT1, LTB, TRAC, TRBC1, MAP4K2, IL7R, VILL, SIRPG, CD27, DPP4, FAIM3, ACAP1, LAT, RASGRP2, ANKRD55, LEF1, LIME1, FLT3LG |
| T cells CD4 memory  resting | GZMM, CD6, CD96, TRAV13-1, DGKA, PTGER2, CTSW, ICOS, GPR171, ZAP70, TRAV13-2, ZFP36L2, BCL11B, PBXIP1, TRAC, SH2D1A, GZMA, CD28, TRAV8-6, GZMK, CD7, CD2, CD40LG, RPL10L, LEF1, KLRB1, CD3D, NKG7, EPB41, IL7R, SIRPG, CD3G, RPL3P7, CD69, RASA3, FLT3LG, CTLA4, CD247, LCK, TCF7, RCAN3, UBASH3A, TRAV9-2, LTB, TRBC1, RASGRP2, CCR6, ITK, CD4, TRAT1, ETS1, CD27, FBXL8, ST8SIA1, TRAV21, DPP4, FAIM3, CCL5, GPR25, PTPRCAP, CD3E, LY9, LIME1, GRAP2 |
| T cells CD4 memory  activated | GPR171, RRP9, SKA1, IL12RB2, TNFRSF4, NKG7, TNIP3, IL2RA, DPP4, GPR19, CDC25A, TRAT1, ICOS, IL4, LAG3, SH2D1A, CD28, CSF2, TRAC, CD3D, IL9, CD40LG, LCK, GZMB, CXCL13, IL26, CD7, IL17A, IL3, CTLA4, IFNG, CD2, UBASH3A, CD3G, ORC1, LTA, PMCH, CCL20, CD247, CD6 |
| T cells gamma delta | SKAP1, GPR18, BFSP1, CD8B, KRT18P50, TARDBPP1, BRSK2, LAG3, CCR5, GNLY, SH2D1A, SCN9A, GZMK, LY9, CD160, GYPE, CD244, MAP4K1, UBASH3A, KLRD1, ZNF442, PVRIG, CD300A, SIRPG, CDH12, LHCGR, TCF7, CCL5, TRBC1, CD3D, KLRK1, COLQ, SIT1, KLRG1, GZMM, LAT, GZMA, VNN2, DUSP2, LCK, CST7, GZMH, GFI1, KLRB1, IL18RAP, CD8A, MAGEA11, CD2, PLEKHG3, GPR171, CD3G, PTGDR, CXCR6, PRF1, TRDC, GZMB, CD247, IL2RB |
| TIL | GIMAP7, IL2RG, LAT, CFHR1, CD40, CCR7, 6-Sep, SIT1, PIK3CD, ARHGAP9, IL2RB, TCL1A, TNFRSF4, CCL5, GVINP1, CD79A, GPR171, NCF4, PLAC8, MZB1, ICOS, GIMAP5, ITK, P2RY8, PARVG, PVRIG, GIMAP6, LAX1, NLRC3, DOK2, CD247, ACAP1, CCR2, LCP2, PRKCQ, F5, KLRK1, IL10RA, MS4A6A, TBX21, CSF2RB, CD28, FAM65B, MAL, GPR18, CD48, CD3D, PAX5, KLRD1, CD27, CD3E, LCK, CD6, SELPLG, IFFO1, IRF4, VAMP5, CD52, CD38, CD2, SH2D1A, SIRPG, CD3G, TIGIT, SELL, GPSM3, CD86, PAG1, CYBB, XCL1, GZMK, PTPRC, DOCK11, SASH3, GLYR1, IL16, SLAMF1, XCL2, ITGA4, ARHGAP30, TRAF3IP3, CST7, PLEK, FCRL3, na, CORO1A, GIMAP4, IKZF1, DOCK2, FCRL5, IL7R, ARHGAP25, NCKAP1L, THEMIS2, STAT4, CD8A, KLHL6, ARHGAP15, FYB, LY9, HCST, MFNG, CD53, PTPRCAP, TRAT1, TARP, SPNS1, INPP5D, SLAMF6, HCLS1, LILRB1, STK10, CLEC2D, PRKCB, MS4A1, ITM2C, LPXN, TBC1D10C, EVI2B, CFH, MPEG1 |
| Cytotoxic cells | RORA, APBA2, KLRK1, GZMA, GZMH, SIGIRR, KLRD1, GNLY, RUNX3, NKG7, DUSP2, KLRB1, WHDC1L1, ZBTB16, CTSW, GZMK, KLRF1, APOL3 |
| NK CD56dim cells | KIR3DL1, GTF3C1, FLJ20699, KIR2DS5, KIR2DS2, KIR2DL3, EDG8, TMEPAI, KIR3DS1, KIR3DL2, KIR2DS1, GZMB, SPON2 |
| NK CD56bright cells | FOXJ1, MADD, MPPED1, MUC3B, XCL1, BG255923, NIBP, DUSP4, PLA2G6, RRAD |
| NK cells  resting | GNLY, BPI, CAMP, CD96, PRR5L, TXK, CDHR1, GZMH, CEACAM8, NKG7, TRBC1, TTC38, IL18RAP, CD160, DEFA4, NAALADL1, ELANE, CCL5, IL2RB, CTSW, PTGDR, IL18R1, PVRIG, KIR2DL1, S1PR5, KLRD1, CD244, GZMB, LCK, KLRC3, KIR3DL2, GZMK, PLEKHF1, KLRB1, PRF1, GFI1, ZNF135, PTPRCAP, GZMM, CD7, IL12RB2, MS4A3, KLRK1, NME8, ZAP70, TBX21, CD2, TEP1, GZMA, SH2D1A, KLRF1, MGAM, KLRC4, AZU1, TRDC, CD247, CST7 |
| NK cells  activated | KLRK1, TXK, GPR171, CTSW, CCL4, KIR2DS4, GPR18, PRF1, DPP4, IL18R1, KLRB1, KIR2DL4, NKG7, CD96, LTB, NCR3, LTA, CST7, PVRIG, KIR3DL2, SOCS1, IL18RAP, CD69, CCL5, CSF2, CCND2, PTGDR, CD7, GZMA, SH2D1A, NAALADL1, IFNG, GZMH, APOL6, GZMB, CD244, PTPRCAP, OSM, KLRF1, IL2RB, KLRC3, KIR2DL1, PTGER2, TRDC, APOBEC3G, IL12RB2, CDK6, CD247, FASLG, TNFSF14, GNLY, GZMM, S1PR5, GRAP2, PRR5L, TBX21, ZAP70, LCK, KLRD1 |
| DCs resting | NCF2, HLA-DQA1, MMP12, C1orf54, PPFIBP1, ACP5, TMEM255A, CD1B, TREM2, CD1C, CLEC7A, CCL13, AIF1, DHRS11, SLAMF8, FZD2, CCDC102B, RNASE6, ALOX15, CD68, EGR2, CLEC10A, CLEC4A, PLA2G7, SCN9A, CD1A, CCL18, CCL17, CD1E, CCL22, FCER2, SLC15A3, CD209, FAM198B, FLVCR2, FCER1A, FPR3, CD33, CLIC2, IGSF6 |
| DCs  activated | EBI3, CYP27A1, TNFRSF4, HESX1, SLCO5A1, CCL20, SIGLEC1, ARHGAP22, KYNU, CCL22, CXCL11, CCR7, BIRC3, MAP3K13, CCL1, FPR3, PLA2G7, PLA1A, IL2RA, CD80, CD1B, CD86, ST3GAL6, HTR2B, CCL19, CD1E, CCL18, RASSF4, CHST7, TMEM255A, NR4A3, CCL17, CCL5, CXCL10, IFI44L, CCL13, IDO1, LAMP3, IL12B, PDCD1LG2, MSC, DHX58, TNFRSF11A, SLC15A3, MMP12, TREM2, SLC2A6, ETV3, RSAD2, TNFAIP6, PTGIR, CLIC2, CCL8 |
| iDCs | BLVRB, PPARG, TM7SF4, FABP4, LMAN2L, CSF1R, GUCA1A, CLEC10A, CD1C, NM_021941, MS4A6A, RAP1GAP, HS3ST2, CTNS, F13A1, CD1E, SYT17, GSTT1, CH25H, PREP, MMP12, NUDT9, TACSTD2, SLC7A8, SLC26A6, CD1B, CARD9, CD1A, FZD2, VASH1, ABCG2 |
| aDCs | CD83, INDO, OAS3, CCL1, LAMP3, EBI3 |
| pDCs | IL3RA, CLEC4C |
| Macrophages M0 | CCL18, MMP9, CCL22, QPCT, IGSF6, GPC4, CXCL5, ACP5, NCF2, SLC12A8, CSF1, VNN1, CCL7, CYP27A1, TNFSF14, CD68, TREM2, BHLHE41, SLC15A3, COL8A2, DCSTAMP, PPBP, HK3, PLA2G7, SLAMF8, CHI3L1, MARCO, AQP9, ADAMDEC1, FAM198B, CXCL3, C5AR1, CCDC102B |
| Macrophages M1 | IL2RA, IFI44L, KIAA0754, CLIC2, KYNU, PTGIR, LAMP3, CXCL9, LILRA3, CCL8, APOL3, PLA1A, TRPM4, RASSF4, CCL5, LILRB2, DHX58, CD38, RSAD2, TLR7, SIGLEC1, CXCL13, ARRB1, TLR8, ADAMDEC1, EBI3, SLAMF1, SOCS1, NOD2, CCR7, CCL19, CXCL11, APOBEC3A, CXCL10, CYP27B1, TNFAIP6, TNIP3, CD40, AQP9, APOL6, CHI3L1, HESX1, SLC2A6, GGT5, IDO1, ACHE, SLC15A3, LAG3 |
| Macrophages M2 | CFP, WNT5B, CD209, CLEC4A, FRMD4A, CLIC2, CCL14, CD4, FAM198B, EBI3, HRH1, P2RY13, SIGLEC1, FES, ALOX15, CD68, CHI3L1, HTR2B, TREM2, GGT5, MS4A6A, CCL13, CRYBB1, CCL8, CLEC10A, GSTT1, FZD2, CCL23, SLC15A3, CCL18, TLR8, PDCD1LG2, NPL, RENBP, NME8, ADAMDEC1, AIF1 |
| Mast cells  resting | ATP8B4, STXBP6, TPSAB1, LTC4S, BMP2K, NOX3, BPI, HDC, CTSG, STAP1, CEACAM8, P2RX1, CPA3, ST8SIA1, C3AR1, FAM124B, RGS13, SLC12A8, PAQR5, ADAMTS3, MS4A3, CMA1, FAM174B, MYB, GFI1, MS4A2, NTRK1, CLC, HPGDS, CD33, P2RY14, IL18R1, RAB27B, PRG2, 8-Sep, CRISP3, FCER1A, ADRB2 |
| Mast cells  activated | MS4A2, 3-Mar, TEC, GZMB, MYB, IL3, MS4A3, CCL1, STXBP6, CPA3, RGS13, IL1A, PRG2, CLC, NOX3, TPSAB1, CMA1, SLC12A8, IL1B, HDC, ATP8B4, P2RX1, CCL20, CCL4, BPI, CSF2, HOXA1, NTRK1, CXCL3, CD33, CTSG, IL18R1, IL5, IL1RL1, LINC00597, RAB27B, FCER1A, HPGDS |
| Neutrophils | FLJ11151, CDA, VNN3, EVI2B, FPR1, CHI3L1, HSD17B11, S100A12, PADI4, EMR2, KDM6B, CEACAM3, MEGF9, CD93, MAK, TNFAIP6, MME, DPEP2, CXCR1, LST1, TLR2, SLC25A37, P2RY14, REPS2, NLRP12, FCAR, IL8RA, FCGR3B, CREB5, HSPA6, ALPL, FFAR2, TLR8, SLC22A4, CASP5, LILRB2, CSF3R, SELL, IL18RAP, BST1, TNFRSF10C, DYSF, FAM212B, MNDA, TRANK1, HIST1H2BC, P2RY13, IL8RB, FPR2, EMR3, SIGLEC5, STEAP4, TREML2, LILRA2, QPCT, KCNJ15, G0S2, CLC, NCF2, CRISPLD2, MGAM, PLEKHG3, GPR97, MXD1, IGSF6, VNN2, AIF1, FPRL1, CFP, AQP9, PDE4B, MMP25, APOBEC3A, C5AR1, PGLYRP1, VNN1, CHST15, TREM1, MEFV, NFE2, KIAA0329, CAMP, BTNL8, CYP4F3, HPSE, CCR3, CXCR2, HAL |
| Eosinophils | HRH4, P2RY2, IL18R1, FFAR2, BPI, MYO15B, ZNF222, CD244, BANK1, MS4A3, RGS13, DAPK2, EMR2, ACACB, RGS1, SMPD3, TIPARP, KBTBD11, CLC, P2RY13, DPEP2, IL1RL1, THBS1, PKD2L2, C5AR2, GIPR, THBS4, IL5RA, P2RY14, REPS2, TKTL1, C3AR1, IL18RAP, CAT, HES1, RNU2, TREML2, RCOR3, ST3GAL6, HIST1H1C, RNASE2, GALC, SYNJ1, ABHD2, BCL2A1, RRP12, EMR1, SAMSN1, MXD1, CCR3, TGIF1, IGSF2, NCF2, LRP5L, PADI4, MGAM, PLEKHG3, DACH1, DEPDC5, EPN2, P2RY10, EMR3, GPR183, GPR65, C9orf156, ALOX15, GPR97, LRMP, MAK, PDE6C, SMPDL3B, SIAH1, TRPM6, GPR44, KCNH2, CYSLTR2 |
